# Atrial resynchronization: an overlooked concept in heart failure and conduction system pacing: review of selected literature with emphasis on atrial anatomy

**DOI:** 10.1093/europace/euag088

**Published:** 2026-06-05

**Authors:** Atul Prakash, Richard Sutton, Damián Sánchez-Quintana, Robert H Anderson

**Affiliations:** Department of Cardiology, St Mary’s Hospital General Hospital & Rutgers University, Passaic, New Jersey 07055, USA; Department of Cardiology, Hammersmith Hospital Campus, National Heart & Lung Institute, Imperial College, London, UK; Department of Human Anatomy and Cell Biology, Faculty of Medicine and Health Sciences, University of Extremadura, Badajoz, Spain; Biosciences Division, Newcastle University, Newcastle upon Tyne, UK

**Keywords:** Atrial anatomy, Atrial conduction, Atrial stimulation, Atrial resynchronization

## Abstract

**Aims:**

Unlike ventricular electrical disease, the impact of similar atrial disease on cardiac function has received insufficient importance. We aimed to examine data on atrial anatomy, electrical disease, and pacing to ameliorate abnormalities, offering pointers towards future practice.

**Methods and results:**

We used PubMed to explore atrial anatomy, activation, and pacing aiming to improve function. From inception of pacing, causes of deterioration in ventricular systolic function went unrecognized. Their eventual recognition led to fresh pacing strategies, termed conduction system pacing, which have yielded improved and preserved long-term ventricular function: now standard practice. Study of atrial anatomy/conduction implies that similar approaches may improve atrial function and also reduce incidence of atrial fibrillation. Impact of atrial electrical disease on diastolic and subsequent ventricular systolic function has been ignored, despite data showing how pacing alternative atrial sites can abbreviate atrial activation and improve ventricular filling: methods that have not been widely adopted. Comprehension of atrial anatomy/conduction underpins abandonment of right atrial appendage pacing paralleling the move away from right ventricular apical pacing on similar principles.

**Conclusion:**

When treating heart failure, atrial resynchronization should be considered along with ventricular resynchronization. Crucially, convenient pacing sites have been outmoded by active-fixation leads that can be placed at stimulation sites approximating normal conduction, including the atria; achievement hinges on understanding atrial anatomy and activation. Atrial resynchronization may significantly improve cardiac function where dyssynchrony relates to atrial disease/conduction delay, but more evidence of benefits must be collected. As resynchronization therapy moves forward, there should be parallel focus on evaluating coordination of atrial activation and timing with respect to the ventricles.

## Background

Restoration of optimal atrial function, particularly regarding activation of, and conduction through, the atrial walls, has thus far received scant emphasis in the context of treatment of heart failure. The lack of emphasis on the influence that atrial function may play in those with heart failure largely stems from early studies that failed to show any advantage of rhythm control in maintaining adequate atrial function when compared with simple rate control.^1^ Those demonstrating the beneficial impact of rhythm control, by maintaining sinus rhythm in patients with systolic heart failure, subsequently focused attention on the importance of preservation of atrial function.^2^ This need to preserve adequate atrial function has now increasingly become recognized, with further data showing the success of rhythm control in improving systolic heart failure.^3,4^ Atrial function and atrial arrhythmias should now be seen in the context of atrial cardiomyopathy which has recently been the subject of a major consensus document.^5^ The definition of atrial cardiomyopathy is restated therein as ‘Any complex of structural, architectural, contractile or electrophysiological changes affecting the atria with the potential to produce clinically relevant manifestations’. Clearly this definition encompasses most or all of the patients considered in this review, especially the elderly, and the majority will be in Stage 2 or 3 of the consensus classification. A thrust of the consensus is to emphasize early recognition and preventive treatment. However, device therapy such as atrial pacing is not mentioned.

The need to restore atrial function in the setting of diastolic failure, in contrast, is still not fully appreciated.^6^ In this respect, it is well accepted that abnormal ventricular activation can adversely affect cardiac function.^7^ Abnormal atrial activation, and its contribution to ventricular filling, is less well understood. In this review, therefore, we address the impact of abnormal atrial activation and conduction in the setting of heart failure. We discuss potential correction by atrial resynchronization, making comparisons with the known advantages of ventricular resynchronization therapy. Here, in the pacing of the ventricles, the importance of the specialized atrioventricular conduction system is fully appreciated. Such a specialized system is not to be found within the atrial walls, although the nodes that produce the sinus impulse, and slow it prior to ventricular activation, are components of the atrial walls.^8^ We begin our review, therefore, by providing an account of the anatomy and structure of the atrial walls. Such information is becoming more important in the light of the burgeoning interest in the interatrial conduction pathway known as Bachmann’s bundle.^9,10^ We then show how such morphological knowledge can underscore appropriate therapeutic options for atrial resynchronization. This review proposes that atrial resynchronization through anatomically informed pacing can mirror benefits seen in ventricular resynchronization, potentially transforming current clinical approaches.

## Historical and recent evidence of atrial anatomy and mural architecture relative to atrial resynchronization

**Figure 1:**
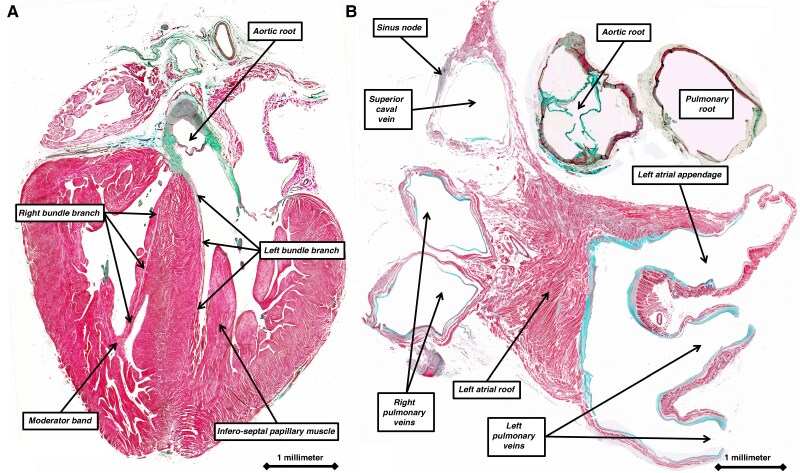
The histological sections, stained using the trichrome technique, show the differences between the substrates underscoring ventricular (*A*) as opposed to atrial (*B*) activation. Panel *A* shows a ‘four-chamber’ section through the ventricular walls of a human foetus at 22 weeks of gestation, demonstrating the insulated nature of the right and left bundle branches. Panel *B*, a short-axis section across the atrial chambers of an adult human heart, shows the location of the sinus node. Conduction from the node, however, depends on the passage of the cardiac impulse through the working atrial cardiomyocytes, with the alignment of the aggregated cardiomyocytes underscoring preferential conduction.

It has been specific pacing of the ventricular conduction system that has underscored the approach to ventricular resynchronization. The arrangement of the anatomical system that ensures normal ventricular activation was revealed by Tawara^11^ over 100 years ago. Shortly thereafter, Keith and Flack^12^ demonstrated the presence, in the walls of the right atrium, of the cardiac pacemaker. In the decade that followed, leading up to the description of the interatrial bundle described by Bachmann,^9^ it was shown that, unlike the insulated bundle branches responsible for ventricular activation (*Figure 1A*), it was the aggregation of working atrial cardiomyocytes in parallel fashion that underscored preferential conduction through the atrial walls (*Figure 1B*). To discover his bundle, Bachmann had experimented on dog hearts. He noted that ‘a distinct band of muscle tissue stretches from the right auricle to the base of the left auricular appendage and forms apparently the most direct connection between the auricles’.^9^ Having crushed the bundle, he noted that the interatrial conduction time was ‘from 3 to 4.6 times greater than the normal average’. Bachmann had cited the previous work of Lewis, Meakins, and White,^13^ who had called attention to ‘the straight course taken by the fibres of this muscle band’. Neither Bachmann nor Lewis, Meakins, and White, however, had provided histological images to illustrate this feature. Lewis, Meakins, and White, nonetheless, when emphasizing the ‘straight course taken by the fibres of this muscle band’, indicated they had noted the importance of the alignment of the myocytes in underscoring a relatively high rate of conduction in the ‘intercaval and band regions’.^13^ These investigators also denied the existence of any rapidly conducting pathways between the sinus and atrioventricular node. The potential existence of such internodal tracts as part of a specific ‘atrial conduction system’ has continued as a controversy through the latter part of the 20th and into the 21st century.^14^ It is worth citing, therefore, the specific passage from the work of Lewis, Meakins, and White. ‘Our belief is that the excitation passes in all directions from the node by similar connections with the surrounding muscular tissue; that it passes with equal facility along all these paths, and that there are no paths which may be demonstrated as of specially high or low conducting power; and that, once in the auricular muscle, the general course of the muscle fibre bands is taken at uniform speeds’.^13^

**Figure 2:**
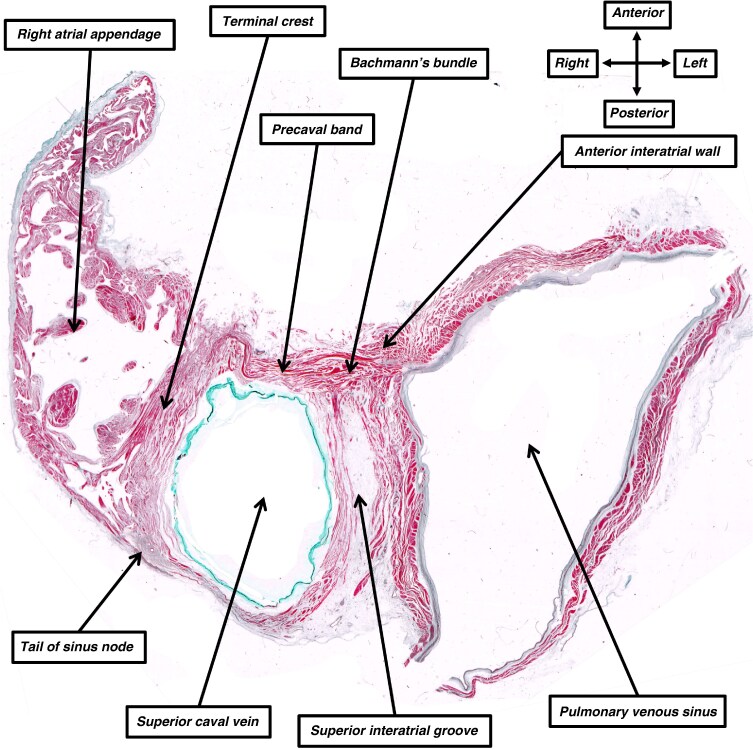
The image shows a histological section, taken in the short axis of an adult human heart, and again stained with the trichrome technique. It shows the parallel aggregation of the working atrial cardiomyocytes in the terminal crest as it crosses, as the precaval band, in front of the orifice, merging with the anterior interatrial wall to produce the area known as Bachmann’s bundle. Note also the parallel aggregation of the cardiomyocytes within the leftward wall of the superior caval vein. This also merges with the area of Bachmann’s bundle. This part of the superior cavoatrial junction forms the rightward wall of the superior interatrial groove. It is also possible to recognize the tail of the sinus node positioned subepicardially within the terminal groove.

Our own histological investigations support the conclusions reached by Lewis, Meakins, and White (*Figure 2*), although we recognize that the function of a particular cardiomyocyte cannot be determined simply by examining it with the microscope. The histological sections show that the working unit of the atrial walls, as with the ventricular walls, is the cardiomyocyte. It is not possible to detect, from the sections, any coalescence of the individual cells that justifies the description of a myocardial ‘fibre’. The arrangement of the cardiomyocytes within their supporting fibrous matrix does reveal a preferential alignment that supports the notion of anisotropic conduction (*Figure 2*). This dictates that the speed of conduction is greatest along the long axis of the chains of cardiomyocytes, and least perpendicular to the long axis.^15^ In this regard, when assessing the pattern of grouping of the individual working cardiomyocytes, a direct pathway can be traced from the terminal crest, through the precaval band, and into the cranial part of the anterior interatrial wall. It is the latter area that is recognized as Bachmann’s bundle (*Figure 2*). The individual cardiomyocytes within the right and left walls of the superior interatrial groove are similarly aggregated in parallel fashion. This arrangement favours preferential conduction across the cranial rim of the oval fossa towards the origin of Bachmann’s bundle (*Figure 2*).

When examining the histological sections with the light microscope, even if not possible to predict their function, it is possible to distinguish between the cardiomyocytes making up the sinus node and those within the remainder of the atrial walls (*Figure 1B*). The alignment of the individual cardiomyocytes within the walls produces the ‘grain’ shown by removing the epicardial lining of the walls (*Figure 4*). It is not possible, however, to observe individual components segregated within the walls, such as the alleged ‘septopulmonary’ and ‘septoatrial’ bundles. These components are no more than areas in which the individual cardiomyocytes are grouped together to show a particular alignment. The walls themselves are contiguous entities made up of working cardiomyocytes, although the manner of aggregation is not uniform throughout the thickness of the walls (*Figure 1B*).

**Figure 3:**
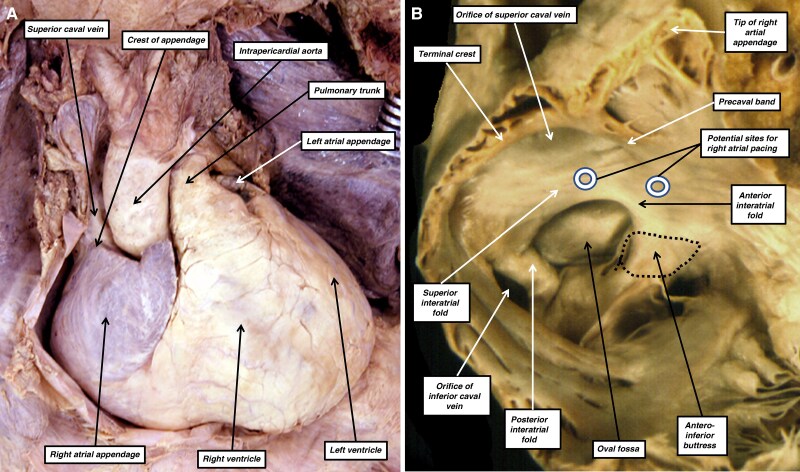
Panel *A* shows the heart as it lies within the chest having reflected the anterior pericardial covering. It can be seen that the appendage forms the entirety of the anterior wall of the right atrium. Panel *B*, from a different heart, has been prepared by removing the anterior component of the appendage, along with the parietal part of the right atrioventricular junction. It shows how the systemic venous tributaries open into the right atrium posteriorly, with the site of the oval fossa also being seen on the posterior and rightward wall of the chamber. Note how the terminal crest passes in front of the orifice of the superior caval vein as the precaval band; it then merges imperceptibly with the superior component of the anterior interatrial wall. The anteroinferior part of the interatrial wall is the second atrial septum, which becomes contiguous with the vestibular myocardium. We have marked two sites where, based on our interpretation of the mural architecture, pacing in the right atrium would activate Bachmann’s bundle. These are the merge point of the precaval band with the anterior interatrial wall and the superior rim of the oval fossa.

**Figure 4:**
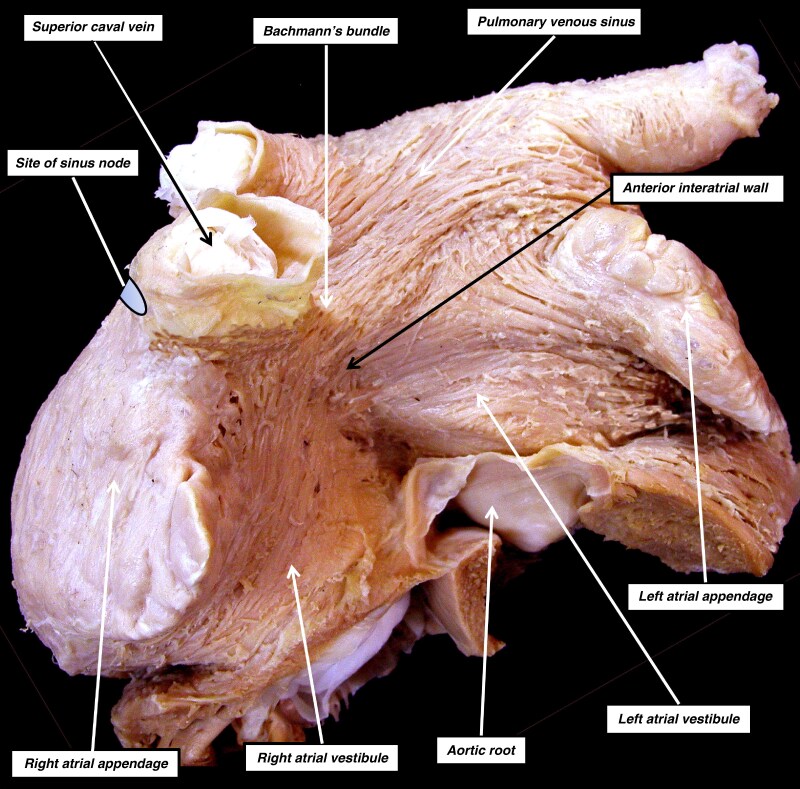
The image shows the atrial chambers of an adult heart viewed from the front, having removed the chambers from the ventricular mass at the level of the atrioventricular junctions, retaining the proximal parts of the ventricular walls and the basal component of the aortic root. The epicardial covering of the anterior interatrial wall has been removed to show the ‘grain’ of the aggregated working atrial cardiomyocytes that make up the atrial walls. The site of the sinus node has been marked in the terminal groove. It is lateral and inferior to the crest of the right atrial appendage. The area known as Bachmann’s bundle is the superior part of the interatrial groove. It is continuous on the endocardial aspect with the merging point of the precaval band of the terminal crest (see *Figure 2*). Note the parallel ‘grain’ of the aggregated cardiomyocytes in the cranial part of the anterior interatrial groove. The parallel arrays continue into the roof of the pulmonary venous sinus of the left atrium and extend to the mouth of the left atrial appendage.

**Figure 5:**
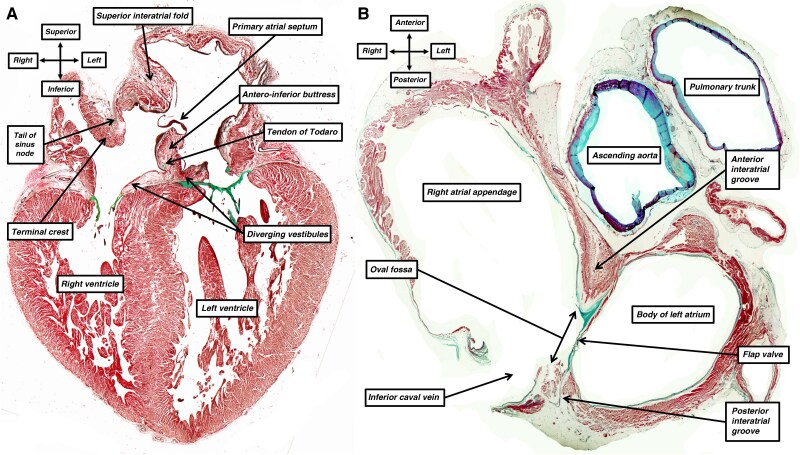
The histological sections show the make-up of the atrial septal components. Panel *A* is a frontal section from a 36-week human foetus. It shows how the primary atrial septum has become fibrous. It apposes on the left side of the superior interatrial fold and in postnatal life will fuse with the fold to close the oval foramen. The primary septum is itself anchored to the atrioventricular junctions by its anteroinferior buttress. The buttress is then contiguous with the atrial vestibules, which diverge to form the atrial boundaries of the inferior pyramidal space. Panel *B* is a short-axis section through the middle of the oval fossa in an adult heart. The primary septum can be seen forming the floor of the fossa. In the foetal heart, it formed the flap valve of the oval foramen. The section of the adult heart shows that the anterior and posterior rims of the fossa are infoldings of the atrial walls. Note the adjacency of the right atrial part of the fold to the ascending aorta.

On the basis of the histological findings, therefore, coupled with the dissections, it is reasonable to presume that the pathway from the sinus node, through the precaval bundle and Bachmann’s bundle, provides optimal activation of the walls of the left atrium (*Figures 3* and *4*). An alternative pathway might use the posterior rim of the oval fossa.^16^ The right atrial endocardial origin of Bachmann’s pathway, nonetheless, is likely to be the optimal site for pacing.^17,18^ This site is also part of the anterior interatrial groove. As such, it is close to the intrapericardial aorta, and the atrial wall is relatively thin at this site (*Figure 5*). This feature is worthy of attention for those inserting active-fixation leads optimally to pace the atria. Monitoring of the electrogram from the lead tip for current of injury and subsequent repolarization will give the necessary information to avoid perforation into the aorta.

Our findings indicate that an appreciation of the overall atrial anatomy provides the basis for understanding how the impulse activating atrial contraction is conducted through the atrial walls. The source of the cardiac impulse, the sinus node, is an integral component of the right atrial musculature.^8^ The left atrial chamber, in contrast, lacks a specific pacemaker. Its walls must, therefore, be activated by interatrial pathways. These features are just as pertinent to atrial resynchronization as they are to normal atrial activation. It is reasonable to presume that pacing at one or more of the major pathways for normal interatrial conduction will provide the optimal sites for resynchronization of diseased atrial walls.

This requires, therefore, an understanding of the extent of the pathways which, in the normal heart, provide direct connections between the myocardial walls of the right and left atrial chambers. When assessing these connections in the clinical setting, the first step is to ensure that the heart is described as viewed as it lies within the chest; this arrangement is now termed the attitudinal approach by anatomists. When using this approach, it can be seen that the triangular right atrial appendage forms the entirety of the anterior wall of the right atrium (*Figure 3A*). The terminal crest is then positioned vertically within the right atrium. It forms the rightward border of the posteriorly located systemic venous sinus, which cranially and caudally receives the superior and inferior caval veins (*Figure 3B*). The terminal crest itself, when traced cranially, extends in front of the orifice of the superior caval vein as the precaval band.^19^ Having crossed in front of the superior cavoatrial junction, it merges on the leftward wall of the atrial chamber with the anterior interatrial wall, which forms the anterior margin of the oval fossa. The fossa marks the site, during development, of the oval foramen. The floor of the fossa is made up of the primary atrial septum, which is anchored to the atrioventricular junctions by the prominent anteroinferior septal buttress (*Figure 5A*). Although initially myocardial, already by the end of the foetal period, when it provides the flap valve of the oval foramen, the primary septum has largely become a fibrous entity. The rims of the foramen then provide the major myocardial pathways between the right and left atrial chambers. The rightward rim of the superior interatrial groove, furthermore, merges with the precaval band as it forms the anterior interatrial wall (*Figure 3B*). Additional myocardial connections are then to be found between the walls of the coronary sinus and the left atrium in the left atrioventricular groove.^20^ These strands, however, are unlikely to be involved in the normal activation of the left atrial myocardium from the sinus node.

It is still frequently suggested that the cranial rim of the oval fossa is the second atrial septum, or the ‘septum secundum’. This is not true.^21^ It had been shown by the end of the 19th century that the cranial rim of the fossa was an infolding between the attachments of the pulmonary veins to the left atrium, and the attachment of the systemic venous sinus to the right atrium.^22^ As is the case with the superior rim (*Figure 5A*), the anterior and posterior rims of the fossa are also folds in the atrial walls (*Figure 5B*). To emphasize again, the right atrial rim of the superior fold merges with the precaval bundle to form the anterior interatrial groove. The posterior rim is yet another fold that can provide a pathway for interatrial conduction. Additional strands can be found crossing the folds that form the rims of the fossa.^23^ It is the continuity between the infolded walls around the fossa, nonetheless, which provides the predominant interatrial pathways.

Although the infolded rims are not true septal entities, there is a true second atrial septum. This is the anteroinferior buttress. It anchors the floor of the fossa, which functioned as its flap valve prior to postnatal closure, to the atrioventricular junctions (*Figure 5A*). The buttress is formed by muscularization of the mesenchymal components that, during development, closed the primary atrial foramen.^24^ The myocardium produced by muscularization also provides the fast pathway into the atrioventricular node.^25^ The buttress itself merges with the vestibules of both the right and left atrial chambers. It thus provides a direct pathway for conduction between the atrial vestibules. This vestibular pathway is the caudal part of the prominent anterior interatrial wall, with the groove in the wall forming the anterior rim of the oval fossa (*Figure 3B*). Taken overall, as assessed anatomically, it remains the cranial part of the anterior rim of the oval fossa which provides the major pathway for interatrial conduction. This is because the precaval band, having passed in front of the orifice of the superior caval vein, merges with the rightward rim of the superior interatrial fold to form the anterior interatrial wall. And it was this pathway that was identified by Bachmann as the major route for activation of the left atrial myocardium.^9^

## Differences between atrial and ventricular conduction

The area making up Bachmann’s bundle is anatomically different from the atrioventricular conduction axis, initially identified by Tawara,^11^ and usually described by electrophysiologists as the His–Purkinje system. Bachmann’s bundle is composed of working atrial cardiomyocytes arranged in a parallel fashion, which permits more favourable conduction in comparison with the regular cardiomyocytes making up the greater parts of the atrial walls. It is the parallel grouping of such working cells that permits preferential conduction along their long axis. In contrast, the ventricular conduction system is composed of cardiomyocytes specialized by virtue of their insulation from the adjacent working cardiac myocytes.^9^ This arrangement permits much faster electrical conduction than can exist in the atria. Thus, there are clear and indisputable anatomical differences between atria and ventricles, which are reflected in the electrophysiological characteristics of the upper and lower cardiac chambers. In the atria, electrical conduction is preferential, including the activation across Bachmann’s bundle. In the ventricular system, conduction is specialized, and much faster, because of the insulation of the contained cardiomyocytes. All of these considerations are pertinent, since claims continue to be made that conduction in Bachmann’s bundle is also ‘specialized’, implying mechanisms of conduction approaching those seen in the atrioventricular conduction axis.^17,26^ This possibility may have been best covered by van Campenhout *et al*.^27^ stating that ‘Bachmann’s bundle shares electrophysiological properties of both Purkinje and atrial fibres’. In this context, ‘fibres’ should be considered as cardiomyocytes. The atrial cardiomyocytes, however, do not share the anatomical features of the atrioventricular conduction axis because they are not insulated from their working neighbours.^9^

## Physiology and pathophysiology

### Normal ventricular diastolic filling

Physiologically, diastole is divided into four phases: namely, isovolumic relaxation, rapid passive filling, diastasis, and atrial contraction.

*Atrial systole* is the contraction of the left and right atrial walls after electrical activation by conduction across the two chambers. On the electrocardiogram, the *P*-wave correlates with atrial depolarization, which leads to atrial contraction and marks the start of atrial systole. Atrial contraction is visible as an ‘a’ wave on an atrial or central venous pressure waveform. Atrial systole, as indicated above, corresponds to the last phase of ventricular diastole during which ventricular filling is completed. Atrial systole lasts around 100 ms and ends prior to ventricular systole. Despite atrial systole being responsible for a minority of the shift in blood volume, it is a valuable contribution to ventricular filling.

### Delayed atrial activation and its impact on cardiac function

Delayed atrial activation causes atrial structural and electrical remodelling.^5^ These changes are related to fibrotic scarring and dilation. The scarring causes delayed activation, while the dilation is its consequence. These changes inevitably result in atrial tissue that is predisposed to the formation of re-entrant pathways. With scarring and dilation, the contractility of atrial tissue is diminished, which decreases ventricular filling during atrial systole. In pigs, Duong *et al*.^26^ have shown that pacing at Bachmann’s bundle improved haemodynamics compared with pacing at the right atrial appendage. Delayed atrial activation can result in atrial systole occurring after closure of the mitral valve, prompting reversal of blood flow into the pulmonary veins, which is a severe haemodynamic handicap. Lesser degrees of late atrial activation are associated with diastolic mitral regurgitation, with raised left ventricular filling pressures being translated into raised atrial pressures during filling, especially when the rapid filling phase has finished but left atrial systole is delayed.^28,29^ As seen on the electrocardiogram in sinus rhythm, P-wave duration is an approximate measure of atrial depolarization time. Prolonged P-wave duration is known to be associated with delayed intra-atrial and interatrial conduction. P-wave duration, while representing an estimate of total atrial activation time, gives little information as to the site of delay. In an elegant study by Eicher *et al.*,^29^ it was found that, in some patients with heart failure with preserved ejection fraction, interatrial conduction delay was associated with delayed left atrial systole, shortened emptying, decreased compliance, and increased filling pressures.

## Pacing considerations

### Lead placement as a hindrance to pacing physiologically

Pacing the right ventricle was most convenient from its apex as it was easy to reach and usually offered a stable site, especially after the advent of tined leads. Major attention to this was drawn by the DAVID study,^30^ from which the harm of creating dyssynchrony by pacing the right ventricular apex was realized, although the writing was on the wall concerning this harm as early as 1925 in the work of Wiggers.^31^ The full comprehension, though, was slow in percolating through to pacing physicians. It was ultimately solved by a radically different approach provided by sheath-delivered active-fixation leads directed to the His bundle, which was introduced by Deshmukh *et al*.^32^ and Zanon *et al*.,^33^ building on the earlier work of Scherlag *et al*.,^34^ Narula *et al*.,^35^ and others. Before this, efforts were focused on attempting to pace the most delayed lateral and basal sections of the left ventricle via the coronary sinus, combined with pacing the right ventricular apex, in this way providing biventricular pacing. It has taken longer to appreciate that atrial pacing in another convenient position, the right atrial appendage, is potentially harmful, and similar to ventricular pacing from the apex of the right ventricle. The right atrial appendage is convenient because it is easy to reach, and tined leads are stable there. Again, sheath-delivered active-fixation leads offer the opportunity to arrive at any part of the right atrial endocardium.

### Lead placement for pacing Bachmann’s bundle

The exact site to select for pacing Bachmann’s bundle has been identified in detailed studies by Lustgarten’s group using both antegrade and retrograde stimulation.^17,18^ Similar findings have been shown by Subramanian *et al*.^36^ Initial atrial activation studies were done by Lemery *et al*.^37^ and Markides *et al*.,^38^ who confirmed that, in patients with paroxysmal atrial fibrillation, activation of the left atrium was by conduction across Bachmann’s bundle. To achieve appropriate pacing, a position posteriorly near the junction of the superior caval vein and the right atrium appears to be optimal (*Figures 1A*, *1B*, and *3B*). Accurate selection of the site must be determined by the stimulated complex. It should follow the stimulus without delay and generate a complex of normal duration, which will be short compared with the pre-existing pathophysiological one. The P-wave axis will similarly be normal, and of greater amplitude than on the pre-pacing electrocardiogram.^17,18,36^ Less effective positions may be identified by longer duration P waves, with a tendency to produce a superior axis, which was considered in the past to be a left atrial enlargement pattern. The question of a specific Bachmann’s bundle signal is less clear, but complexes can be identified.^18,36^ These should not be equated with a His bundle complex. It has been shown that monophasic action potentials with a plateau suggesting specialization can be found at many sites in the atria, but they can be generated by working atrial cardiomyocytes, and do not contribute to any difference in conduction speeds.^39^ Thus, the finding of a ‘specialized complex’ cannot be used to propose the existence of anatomically discrete ‘bundles’ providing rapid conduction. Attention should also be given to the proximity of the site chosen to the ascending aorta by observation of the lead electrograms to avoid perforation and damage to the aorta.

### Atrial activation studies and impact of pacing at different atrial sites: acute and chronic studies

Pacing of the right atrial appendage has been demonstrated to delay atrial activation and alter regional atrial activation.^39^ This can increase the incidence of atrial fibrillation and negatively impact ventricular filling.^40^ As in the ventricle, different pacing sites in the right atrium have been examined, initially acutely, by Saksena’s group and others,^41–43^ with a view to shortening global and regional atrial activation times. Data have been obtained from studies based on contact and 3D mapping.^38,41,43^ Pacing of the middle part of the rim of the atrial septum and Bachmann’s bundle has shown favourable activation of both atrial chambers and is associated with prevention of atrial fibrillation.^41,42^ Pacing from the mouth of the coronary sinus, and other caudal atrial sites, also does not achieve atrial fibrillation prevention.^41,42^ Dual-site right atrial pacing, combining sites high in the right atrium and at the mouth of the coronary sinus, and bi-atrial pacing, combining sites in the high right atrium with distal pacing in the coronary sinus, have been shown to reduce significantly regional and global activation times and additionally have the potential to prevent atrial fibrillation. These approaches were the first acute attempts, during electrophysiological studies, to shorten atrial activation, improve atrial conduction, and reduce atrial fibrillation, primarily by means of earlier excitation of parts of the atrial myocardium.^41,42^ Much early dual-site atrial pacing for prevention of atrial fibrillation had one of the sites at the right atrial appendage, not Bachmann’s bundle, which may account for the assessment of failure of effective prevention.^41–44^ Later work with pacing of Bachmann’s bundle was encouraging,^45,46^ especially the controlled trial reported by Bailin *et al*.^46^ who showed a benefit for Bachmann’s bundle pacing over right atrial appendage stimulation in terms of 75% freedom from recurrence of atrial fibrillation compared with 47% at 1 year *P* < 0.05, supported by the subsequent review of Padeletti *et al*.^47^

Another aspect related to the induction or prevention of atrial fibrillation is ventricular pacing. This is of particular significance when ventricular pacing is unnecessary. This was first shown by Daubert *et al*.,^48^ and again much later in larger numbers by the second DANPACE study.^49^ Much unnecessary ventricular pacing can be avoided by careful programming allowing natural conduction. The DANPACE group also showed, in their randomized controlled trial,^50^ that minimizing atrial pacing in patients with disease of the sinus node did not reduce the risk of new-onset fibrillation, which supports reevaluating current pacing strategies in its prevention.

Evidence of the haemodynamic benefit was largely a by-product of these studies. The aim at that time was to change the atrial substrate that was facilitating perpetuation of fibrillation.^41–46^ This different approach followed the failure of many atrial pacing algorithms designed to achieve the goal of suppression of the initiation of fibrillation, shortening of global and regional activation, and attenuation of conduction delay encountered by atrial premature beats.^47^ The impact of improvement of atrial activation yielded a haemodynamic benefit.^26,51–53^ This haemodynamic benefit can be compared with that produced in the ventricles by improvement in ventricular activation. These concepts are summarized in *Table1*.

**Table 1:** Electrophysiological and haemodynamic studies relevant to progress in atrial resynchronization

Citation	Patients Age/gender	Type of study/sites of stimulation	Intervention Pacing mode	Follow-up duration	Synopsis
Eicher JC, *et al*. Atrial dyssynchrony syndrome: an overlooked phenomenon and a potential cause of ‘diastolic’ heart failure^29^	Step 1: 7 76y 86% F Step 2: 29 81y 62% F	**Acute study** Step 1: patients with HFpEF showing intra-atrial conduction delay. P-wave >120 ms in Lead II Step 2: CSO pacing to resynchronize conduction in HFpEF with interatrial delay	Pacing mode AAI in an acute study	Acute study Showing improved atrial timing and haemodynamics (by echo)	In HFpEF patients with interatrial conduction delay, delayed LA systole, shortened atrial emptying, decreased compliance, and elevated filling pressures were identified. This form of atrial pacing reduced conduction delay significantly, suggesting atrial resynchronization may improve haemodynamics and warrants further research
Subramanian M, *et al*. Electrogram-guided Bachmann bundle area pacing to correct interatrial block: initial experience, safety and feasibility^36^	36 52y 33% F	**Long-term study of BB pacing** BB area—electrogram-guided placement using a Selectra 3D S-40 sheath and Solia S-60 atrial lead	Atrial pacing from a BB lead in a dual-chamber pacemaker or ICD. Exact mode not specified	Follow-up m 19.7 months	Feasibility and safety of electrogram-guided BB pacing in 32 of 36 patients, achieving significant shortening of P-wave duration demonstrated (148.5 ± 16.1–117.8 ± 19.6 ms). BB capture was confirmed electrophysiologically, with partial or complete rectification of interatrial block in majority. Complications minimal, incl. two lead dislodgments and one impedance rise, suggesting a promising option for IAB. Good results in terms of lead data and stability
Prakash A, *et al*. Regional right and left atrial activation patterns during single- and dual-site atrial pacing in patients with atrial fibrillation^41^	20 66y 40% F	**Crossover** Single-site pacing from HRA, CSO, or DCS. Dual-site: HRA + CSO or bi-atrial pacing HRA + DCS	Crossover comparison of single-site bipolar pacing and dual-site pacing. Additional extrastimulus protocols	AF patients studied in an acute format	Intra-atrial conduction patterns analysed during single- and dual-site pacing. Dual-site pacing gave shorter and more synchronized atrial activation, suggesting potential benefit in reducing AF risk
Prakash A, *et al*. Acute effects of dual-site right atrial pacing in patients with spontaneous and inducible atrial flutter and fibrillation^42^	20 64y 50% F	**Acute study** Dual-site RA pacing: HRA + RA septal or CSO vs. HRA alone. DDDR pacing		Symptomatic AF patients. Acute study. AF suppression assessed by extra-stimulation	Improved global atrial conduction times by dual-site RA pacing main contributor to AF suppression Dual-site superior
Bailin SJ, *et al*. Prevention of chronic atrial fibrillation by pacing in the region of Bachmann’s bundle: results of a multicentre randomized trial^46^	120 70y 54% F	**RCT—medium term** BB region 57 patients (upper interatrial septal) vs. RAA pacing 63 patients	Multicentre comparison of atrial septal BB vs. RAA pacing. DDDR LR 80 ppm	12.6 ± 7.4 months for the BB group; 11.8 ± 8.0 months for the RAA group	Multicentre randomized trial of 120 paroxysmal AF patients. BB pacing shortened P-wave duration and kept 75% free of chronic AF at 1 year vs. 47% in RAA pacing. (*P* < 0.05). BB pacing is safer and more effective in preventing AF progression. No farfield R-wave sensing in BB pacing
Padeletti L, *et al*. Atrial septal pacing: a new approach to prevent atrial fibrillation^47^	NS	**Review** Atrial pacing to prevent AF. Suggests BB as a suitable site for atrial pacing to prevent AF	Previous Padeletti study quoted Atrial pacing from CSO	9+/− 6 months 2 atrial tachyarrhythmias recorded in the Padeletti study	Positive findings suggest reduced atrial conduction delays and improved arrhythmia outcomes. Supports BB pacing
Nielsen JC, *et al*. Atrial fibrillation in patients with sick sinus syndrome: the association with PQ-interval and percentage of ventricular pacing^49^	650	**Retrospective investigation of effect of ventricular pacing in SSS** DDDR patients had 1337 h telemetry available. Vent pacing 66% AF in 24.6%	Sub-study of DANPACE 2		Association of AF with PQ interval >180 ms but not with paced AV interval or VP More ventricular pacing, more AF
Kronborg MB, *et al*. Atrial pacing minimization in sinus node dysfunction and risk of incident atrial fibrillation: a randomized trial^50^	539 73y 50% F	**RCT** Single site: RA location not stated	Two dual-chamber modes compared: DDD LR 40 ppm vs. DDDR LR 60 ppm	No prior persist/perm AF. 2 years remote monitoring for time-to-first AF of >6 min. More syncope in DDD 40	Part of the DANPACE 2 study RCT showed that minimizing atrial pacing in SND patients did not reduce the risk of new-onset AF. Supports reevaluating current pacing strategies in AF prevention
Saksena S *et al*. Prevention of recurrent atrial fibrillation with chronic dual site right atrial pacing^51^	15 68y 40% F	**Long term** **Long-term crossover** **dual vs. single site** HRA + rim of CSO or HRA alone	Crossover comparison HRA + CSO vs. HRA DDDR with mode-switch LR 80/90 ppm UR 130/140 ppm	Symptomatic drug-resistant AF 12 months with 6 months in each pacing mode Dual-site pacing superior	Significantly prolonged arrhythmia-free intervals (89 ± 7 days vs. 14 ± 14 pre-implant; *P* < 0.0001) and reduced AF recurrence compared with single-site pacing (*P* = 0.03). Monitoring by Holter and trans-telephonic system
Prakash A, *et al*. Dual site right atrial pacing can improve the impact of standard dual chamber pacing on atrial and ventricular mechanical function in patients with symptomatic atrial fibrillation: further observations from the dual site atrial pacing for prevention of atrial fibrillation trial^52^	79 66y 42% F	**Long-term** **Crossover** Three pacing modes: 1. HRA (*n* = 36) 2. HRA + RA at CSO or low septal (*n* = 39) 3. DDDR (*n* = 76) AAIR (*n* = 3) or support pacing DDI at 50 ppm or VDI	Clinical trial sub-study of DAPPAF^50^	6 months in each mode in a crossover design	Dual-site atrial pacing (not HRA or support pacing) prevented LV dysfunction (no increase in LV end-systolic volume, LVEF stable) and enhanced left atrial function (peak A-wave velocity and atrial filling fraction) compared with baseline. DAP offers superior haemodynamic benefits over standard pacing methods in symptomatic AF patients requiring dual-chamber pacing
Nagarakanti R, *et al*. Left atrial reverse remodeling and prevention of progression of atrial fibrillation with atrial resynchronization device therapy utilizing dual-site right atrial pacing in patients with atrial fibrillation refractory to antiarrhythmic drugs or catheter ablation^53^	34 68y 35% F	**Long-term study. No comparisons** Dual-site atrial pacing of RAA plus rim of CSO	AAIR or DDDR with long AV delay	Symptomatic paroxysmal. AF patients (53%) and persist AF (47%) Medium term: m 4.5 months (*n* = 39) Long-term: m 37 ± 25 months (7–145 months) (*n* = 34) Assessed by access to device diagnostics Dual-site superior to single	Enhanced electrical synchronization of atria and significantly improved left atrial function (peak A-wave velocity increased from 63 ± 23 to 75 ± 19 cm/s at 4.5 months). Significant reduction in left atrial diameter (from 45 ± 5 to 42 ± 7 mm, *P* = 0.003 over 3 years) LVEF stable. 89% remained in sinus or atrial paced rhythm, showing reverse remodelling and less AF progression
Kantharia BK, *et al*. Effect of different location of atrial lead position on nearfield and farfield electrograms in dual chamber pacemaker-defibrillators^54^	17# 63y 5% F	**Acute study** Comparisons: RAA vs. lateral right atrial wall. Both sites tested intraoperatively in the same patients	Bipolar pacing from RAA or lateral RA wall in a standard DDD pacemaker or Defibrillator. Lead tip used for sensing during SR and induced VF	Acute intraoperative evaluation; no follow-up BB not tested	Real-time nearfield and farfield electrograms recorded in SR and induced VF showed the lateral RA wall lead had significantly reduced farfield R-wave amplitudes, RAA 3.5 ± 4.1 mm vs. 1.7 ± 2.2 mm during VF. Nearfield atrial electrogram amplitudes remained unchanged between the two atrial sites, demonstrating that lateral placement effectively reduces oversensing of ventricular signals without compromising atrial sensing—a promising approach to optimize dual-chamber pacemaker/ICD performance
Cazeau S, *et al*. Multisite pacing for end-stage heart failure: early experience^55^	8	**Initial study of multisite pacing for HF** Synchronous biventricular pacing: right and left ventricular (coronary sinus) leads—atrial sensing in SR pacing but no pacing	End-stage heart failure DDD BiV		In eight end-stage CHF patients with widened QRS, synchronous biventricular pacing acutely increased cardiac index by ≈25%, reduced left-sided filling pressures, and led to sustained clinical improvement in the four survivors (NYHA IV to II); turning off multisite pacing during follow-up worsened haemodynamics Encouraging results
Cazeau S, *et al*. Four chamber pacing in dilated cardiomyopathy^56^	1 Male 54y	RAA LA DCS (Medtronic SP 2188-58) LV epicardial lead on free wall (Medtronic 5071) RV Standard	DDD using a Medtronic Chorus 6234 pacemaker	6 weeks	Severe congestive heart failure (NYHA IV) and LBBB underwent four-chamber pacing using a DDD pacemaker Acute haemodynamic studies demonstrated significant improvements in cardiac output and pulmonary capillary pressure At 6 weeks post-implant, the patient had experienced marked clinical improvement, incl. 17 kg weight loss and NYHA II

Drawn from selected milestone papers in order of their appearance in the text.

Abbreviations: AF, atrial fibrillation; AV, atrioventricular; BB, Bachmann’s bundle; BiV, biventricular; CHF, congestive heart failure; CSO, coronary sinus ostium; DAP, dual-site atrial pacing; DAPPAF, The dual-site atrial pacing for prevention of atrial fibrillation trial^50,51^; DCS, distal coronary sinus (left atrium); DDD, DDDR, AAAIR, AAI, pacemaker codes; HF, heart failure; HRA, high right atrium; ICD, implantable cardioverter defibrillator; LA, left atrium; LBBB, left bundle branch block; LR, lower rate; LV, left ventricle; LVEF, left ventricular ejection fraction; m, mean; NYHA, New York Heart Association; persist, persistent; ppm, pulses per minute; RA, right atrium; RAA, right atrial appendage; RCT, randomized controlled trial; SND/SSS, sinoatrial node disease/sick sinus syndrome; SR, sinus rhythm; UR, upper rate; VF, ventricular fibrillation; VP, ventricular pacing; y, year.

Later work on alternative atrial pacing sites to offer better atrial function, reduced atrial fibrillation, and improved timing of atrial activation in relation to ventricular timing has been shown in numerous small to medium-sized studies and also supported by reviews.^57–61^ Some other studies using other atrial sites as single sites did not show notable benefit in the prevention of atrial fibrillation, but Bachmann’s bundle was not the site selected for pacing.^62,63^

The possibility of farfield R-wave sensing in alternative right atrial pacing sites must be borne in mind, as this would inevitably reduce pacing benefit,^54^ although this seems less likely with Bachmann’s bundle pacing.^46^

### Comparison with ventricular resynchronization

The benefit of ventricular resynchronization is manifest when mechanical dyssynchrony typically results from delay in activation of the lateral and basal parts of the left ventricle in the setting of left bundle branch block.^55^ Both an abnormal activation sequence and an increase in activation times are responsible for mechanical dyssynchrony. Resynchronization addresses this dyssynchrony by early activation of the lateral and basal left ventricle.^64–66^ Resynchronization therapy was initially achieved by biventricular stimulation, where a lead paced the delayed sites of activation via the coronary sinus.^55,64,65^ The significant disadvantage of multiple leads to achieve this objective prompted the development of single-site pacing in the ventricle by His bundle pacing and left bundle branch area pacing.^32,33^ This development has just been reviewed as a debate which has demonstrated all aspects of biventricular pacing vs. conduction system pacing,^67^ as well as pacing of the right ventricular septum to engage superficial parts of the Purkinje network.^68^ These techniques offer an opportunity to minimize the number of pacing leads in the heart. Early excitation of the left atrium can be achieved by pacing Bachmann’s bundle. In addition to the prevention of atrial fibrillation, this approach produced an improvement in both atrial function and ventricular filling.^46,51–53^ Dual-site atrial pacing may still have a role in both goals, atrial function and atrial fibrillation prevention. In this context, it will be mandatory to assess atrial fibrillation prevention using the burden of the arrhythmia rather than simply the presence or absence of atrial fibrillation episodes.^69^ Pacing of the rim of the foramen of the atrial septum, presumably by early access to Bachmann’s bundle, has also been shown to offer haemodynamic improvement.^51–53^

### Future directions

An unanswered question exists for both the ventricles and the atria. Regarding ventricular pacing, does single-site pacing from the area of the left bundle branch result in global reduction of activation time in patients with significant delay in left ventricular activation and pre-existing dyssynchrony of the basal and lateral left ventricular walls, or is that effect achieved only by biventricular pacing? Single-site ventricular pacing has the potential to prevent the deleterious effects of right ventricular pacing, but will this have sufficient impact to alleviate conduction delay in patients with left bundle branch block? The proponents of left bundle area pacing advocate its use, although electrophysiologically this seems not to be logical when there is an existing area of delay in the basal lateral wall, which can only be addressed by pacing from an adjacent site. The significant advantage of left bundle area pacing, however, is the use of a single lead.

The same dilemma exists in the atria, that of single-site pacing at Bachmann’s bundle and the rim of the foramen of the septum vs. dual-site or bi-atrial pacing. A basic case can be made for pacing of Bachmann’s bundle informed by knowledge of atrial anatomy and conduction. Long-term follow-up data, in the form of controlled trials, are needed to establish these modes, both single-site and dual-site atrial pacing, in the repertoire of cardiac resynchronization therapy. At least the technique of placing a lead at Bachmann’s bundle is now well described and technically feasible.^17,18,36,46,57–61^ These conceptual difficulties prompt recall of 1994, when it was felt that ‘true’ physiological pacing could be achieved only by use of a four-chamber pacemaker, as described in a case report by Cazeau *et al*.^56^

## Conclusions

Resynchronization of the atria may significantly improve cardiac function where dyssynchrony is related to important atrial disease and conduction delay, but more evidence of benefits must be collected. As cardiac resynchronization therapy moves forward, there should be a parallel focus on evaluating coordination of atrial activation and timing with respect to the ventricles.

## Data Availability

Data is available upon reasonable request.
